# Pancreatogenic (Type 3c) Diabetes Revealed by Diabetic Ketoacidosis

**DOI:** 10.7759/cureus.74960

**Published:** 2024-12-02

**Authors:** Dhiran Sivasubramanian, Sharan Prasaanth, Adithya Mani

**Affiliations:** 1 Critical Care Medicine, Christian Medical College Vellore, Vellore, IND; 2 Internal Medicine, Coimbatore Medical College and Hospital, Coimbatore, IND; 3 Internal Medicine, Coimbatore Medical College, Coimbatore, IND

**Keywords:** alcohol-induced pancreatitis, chronic calcific pancreatitis, diabetic ketoacidosis (dka), endocrine and exocrine dysfunction, exocrine pancreatic dysfunction, glucagon deficiency, insulin deficiency, pancreatic enzyme replacement therapy (pert), type 3c diabetes mellitus, misdiagnosis

## Abstract

Pancreatogenic diabetes also known as type 3c diabetes mellitus (DM) is a distinct entity often overlooked and misdiagnosed as type 2 diabetes. It results from exocrine pancreatic dysfunction involving both insulin and glucagon deficiencies due to damage to pancreatic beta and alpha cells. This case highlights a 46-year-old male presenting with diabetic ketoacidosis (DKA), a rare but severe complication of type 3c DM. The patient exhibited symptoms of dehydration, metabolic acidosis, and positive urinary ketones, with imaging revealing chronic calcific pancreatitis. The diagnosis was confirmed using established criteria, and management involved intensive insulin therapy for glycemic control and pancreatic enzyme replacement therapy (PERT) to address exocrine insufficiency. Additionally, lifestyle modifications including alcohol and smoking cessation and a tailored high-protein, fat-restricted diet were implemented. A basal-bolus insulin regimen was introduced for long-term control, with regular follow-ups to monitor metabolic and pancreatic health. This report underscores the importance of accurate diagnosis and a multidisciplinary approach to optimize outcomes in type 3c DM.

## Introduction

Pancreatogenic diabetes also known as type 3c diabetes mellitus (DM) includes both structural and functional loss of glucose-regulating hormone (insulin) secretion in the context of exocrine pancreatic dysfunction [1]. The American Diabetic Association classified pancreatogenic diabetes as type 3c, updated in 2022 [2]. It is commonly overlooked and misdiagnosed as type 2 DM, characterized by impaired insulin sensitivity and inadequate compensatory insulin response [1]. Data on type 3c DM suggests it might be more prevalent than commonly believed [3]. Around 80% of cases of type 3c DM are a result of underlying chronic pancreatitis (CP) [4], while others are due to hemochromatosis, cystic fibrosis, pancreatic cancer, pancreatic trauma, pancreatectomy or pancreatic agenesis [5]. Unlike type 1 and type 2 DM, type 3c DM involves both insulin deficiency and impaired glucagon secretion as there is damage to both alpha and beta cells of the pancreas. Diabetic ketoacidosis (DKA) is a rare occurrence in type 3c DM due to impaired glucagon secretion. Still, it can occur in severe insulin deficiency triggered by stress, infection, or poor glycemic control. This report discusses a rare presentation of DKA in a patient with type 3c DM secondary to chronic calcific pancreatitis.

## Case presentation

A 46-year-old male presented to the emergency department with a two-day history of loose stools, with 10 episodes each day, described as rice-watery in nature. He reported abdominal pain along with a cough and generalized weakness for the past week. The patient gave a history of loss of appetite, malaise and unintentional weight loss over the past month. The patient is a known case of systemic hypertension and type 2 DM for the past eight years managed with amlodipine and oral metformin. Past history included chronic alcohol consumption and smoking for 25 years. He had recently started treatment for alcohol withdrawal with chlordiazepoxide. On examination, the patient appeared dehydrated with sunken eyes, lethargic and emaciated. His vitals showed a pulse rate of 96/min, blood pressure of 150/90 mmHg, oxygen saturation of 96%, respiratory rate of 28/min and a random blood sugar of 254 mg/dl. Physical examination revealed pallor, muddy conjunctiva and dry mucosa. Routine blood investigations showed anemia, and an elevated serum alkaline phosphatase (Table 1). The autoantibody panel was negative and urine analysis showed glycosuria and no signs of infection. Urine ketone was positive.

**Table 1:** Routine blood investigation

Parameters	Patient value	Reference value
Hemoglobin	9.5 g/dl	12-15 g/dl
White blood cell (WBC) count	7.72 x 10^3^	4-11 x 10^3^ cells/μL
Platelet count	377 x 10^3^	150-400 x 10^3 ^cells/μL
Serum Urea	17	17-43 mg/dl
Serum Creatinine	0.8	0.72-1.18 mg/dl
Serum Sodium	138	136-146 meq/dl
Serum Potassium	4.0	3.5-5.1 meq/dl
Total bilirubin	2.0	0-1.2 mg/dl
Direct bilirubin	1.0	0-0.3 mg/dl
International normalised ratio (INR)	1.30	0.9-1.20
Aspartate aminotransferase (AST)	39	0-50 U/L
Alanine aminotransferase (ALT)	24	0-50 U/L
Alkaline phosphatase (ALP)	>1080	30-120 U/L
Serum albumin	2.0	3.5-5.2 g/dl
Serum Amylase	64	28-100 U/L
Serum Lipase	38	0-67 U/L

**Figure 1:**
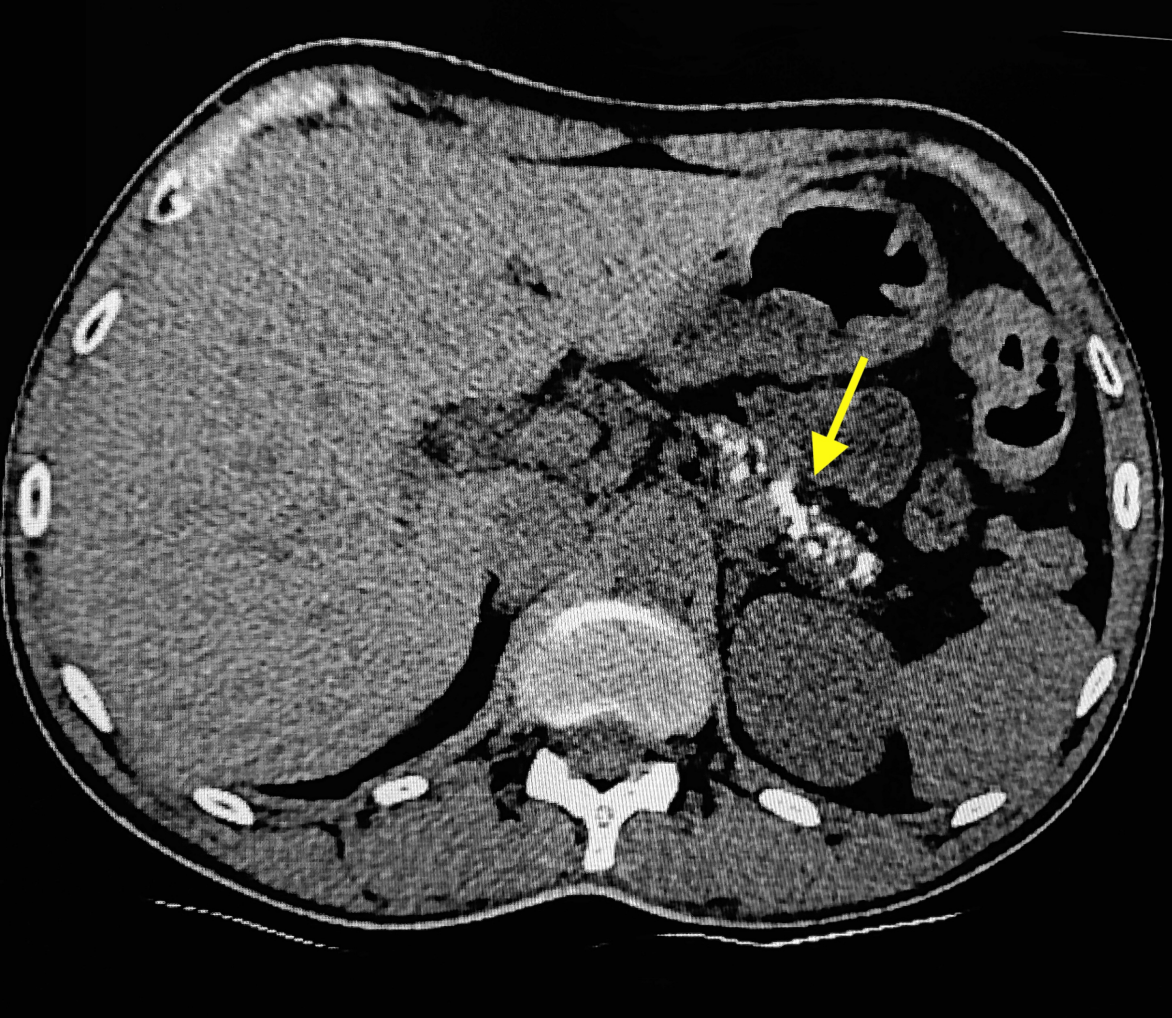
Non-contrast CT of the abdomen showing chronic calcific pancreatitis (yellow arrow). CT: Computed tomography

Ultrasonography of the abdomen showed an atrophic pancreas with parenchymal and intraductal calcifications, common bile duct (CBD) dilatation (1.6 cm), and sludge in the distal CBD. Computed tomography of the abdomen confirmed chronic calcific pancreatitis seen in Figure 1.

The patient was diagnosed with DKA due to symptoms, elevated blood sugar, positive urine ketones and metabolic acidosis. He was started on DKA protocol, intravenous fluids were initiated with isotonic saline to address dehydration, followed by an insulin infusion to manage hyperglycemia. Potassium supplementation was administered to correct hypokalemia, electrolyte levels were monitored closely throughout treatment. The diagnosis of type 3c DM was established based on the imaging findings of chronic pancreatitis and the patient’s clinical presentation with the help of the criteria proposed by Ewald and Bretzel in 2013 [6]. The major criteria include: i) presence of exocrine pancreatic insufficiency, ii) pathological pancreatic imaging, iii) absence of type 1 diabetes mellitus associated autoimmune markers; the minor criteria include: i) impaired beta cell function, ii) no excessive insulin resistance, iii) impaired incretin secretion, iv) low serum levels of lipid-soluble vitamins (A, D, E, and K). All major criteria must be present for diagnosis of type 3c DM.

As the patient stabilized, oral intake was resumed with a high-protein, fat-restricted diet. Pancreatic enzyme replacement therapy (PERT) was introduced to manage exocrine insufficiency and aid digestion. Long-term glycemic control was established using a basal-bolus insulin regimen with 8 IU regular insulin and 10 IU basal insulin. The patient was counseled on the importance of strict alcohol and smoking cessation, adherence to medication, alongside regular follow-up for monitoring his metabolic and pancreatic health.

## Discussion

Type 3c DM accounts for an estimated 1-9% of all diabetes cases and is most commonly caused by chronic pancreatitis [1]. Chronic calcific pancreatitis, the primary etiology in this case involves inflammation-induced glandular fibrosis and atrophy of the pancreas [7]. Chronic alcohol abuse and smoking remain the most common etiological factors [7]. Genetic mutations such as PRSS1 and CFTR gene mutations, pancreatic duct obstruction, and abnormalities in the sphincter of Oddi are also significant contributors [8]. Chronic inflammation leads to progressive loss of both beta and alpha cells. It is often misdiagnosed as type 2 DM due to overlapping clinical features, leading to delays in appropriate management. Unlike type 1 and type 2 DM, type 3c DM involves both insulin and glucagon deficiencies, making it a unique challenge for glycemic control [5].

Diabetic ketoacidosis (DKA), a triad of hyperglycemia, metabolic acidosis, and elevated total body ketone levels [8], is a rare occurrence in type 3c DM. Unlike other types of diabetes, the dual impairment of alpha and beta cells in type 3c DM alters the interactions between key hormones such as insulin, glucagon, cortisol, catecholamines, and growth hormone, which regulate ketone body production, fatty acid oxidation, and lipolysis [5,9]. A notable study by Barnes et al. in 1977 demonstrated that while glucagon plays a significant role in ketogenesis, it is not essential for the development of ketoacidosis [10]. Instead, catecholamines act as compensatory hormones, activating beta receptors to release free fatty acids (FFAs) from triglycerides through lipolysis. FFAs are then utilized in peripheral tissues, contributing to the development of ketogenesis during acute metabolic stress. In type 3c DM, both insulin and glucagon are deficient, creating a complex hormonal environment where catabolic hormones contribute to ketone body production in periods of stress [9,10].

This patient's acute presentation with symptoms of watery stools, loss of appetite, and weight loss within a month initially raised concerns about acute gastroenteritis. Subsequent imaging revealed calcifications in the pancreas, confirming chronic calcific pancreatitis and the positive urine ketone confirmed DKA. The progression from exocrine to endocrine pancreatic insufficiency illustrates the natural history of Type 3c DM.

The management of type 3c DM is challenging, requiring a tailored, multifaceted approach. Insulin therapy is often necessary as the disease progresses as opposed to the oral hypoglycemic drugs used to manage type 2 DM [11]. However, all insulin-based regimens must be approached with caution due to the risk of hypoglycemia. The deficiency of glucagon secretion from islet α-cells makes patients more susceptible to unpredictable episodes of hypoglycemia, even with carefully adjusted insulin doses [11]. Pancreatic enzyme replacement therapy (PERT) is a cornerstone of treatment, improving digestion, promoting glucose tolerance, and enhancing the absorption of fat-soluble vitamins (A, D, E, and K) [11]. Nutritional counseling and the use of PERT are essential for addressing exocrine pancreatic insufficiency.

A comprehensive approach that addresses both endocrine and exocrine dysfunctions is crucial for optimizing outcomes in these patients.

## Conclusions

This case emphasizes the need for increased awareness and recognition of type 3c DM, particularly in patients with a history of chronic pancreatitis or other pancreatic diseases. Misdiagnosis as type 2 DM can delay appropriate treatment and lead to severe complications such as DKA. A multidisciplinary approach involving endocrinologists, gastroenterologists, and dieticians is critical for optimizing the care of these patients. This case serves as a reminder of the importance of considering type 3c DM in patients with diabetes and significant pancreatic pathology.
